# Challenges in implementation of patient‐centred care in cardiac care unit: A qualitative study

**DOI:** 10.1002/nop2.1352

**Published:** 2022-09-04

**Authors:** Firouzeh Charosaei, Shahnaz Rostami, Maryam Esmaeili, Shahram Molavynejad, Zohreh Vanaki

**Affiliations:** ^1^ Department of Nursing, School of Nursing and Midwifery Ahvaz Jundishapur University of Medical Sciences Ahvaz Iran; ^2^ Nursing Care Research Center in Chronic Disease, School of Nursing and Midwifery Ahvaz Jundishapur University of Medical Sciences Ahvaz Iran; ^3^ Nursing and Midwifery Care Research Center, School of Nursing and Midwifery Tehran University of Medical Sciences Tehran Iran; ^4^ Department of Nursing, Faculty of Medical Sciences Tarbiat Modares University Tehran Iran

**Keywords:** cardiac care unit, patient‐centred care, qualitative study

## Abstract

**Aim:**

This study aimed to explore the barriers to the implementation of patient‐centred care (PCC) in the cardiac care unit (CCU) from the perspectives of patients, nurses, physicians and nursing managers.

**Design:**

This study was performed with a descriptive qualitative study approach.

**Methods:**

In this study, the data were collected through face‐to‐face in‐depth semi‐structured interviews with 10 cardiac care nurses, one assistant nurse, two cardiologists, seven patients admitted to the CCU and nine nursing managers and analysed by Graneheim and Lundman content analysis method.

**Results:**

After analysing the data, eight subcategories and three main categories were extracted. The main categories included *challenges related to*: organization, healthcare providers and patients. This study demonstrated the barriers to the implementation of PCC in the CCU. Insights into these barriers can guide interventions aimed at improving the quality of PCC in the CCU, which in turn can lead to improved disease outcomes.

## 1 INTRODUCTION

The quality of nursing care has always been one of the challenges in the healthcare system, especially in the cardiac care unit (CCU), where the quality of care is an indicator for maintaining and promoting the health of patients (Lyman et al., 2017). Patients admitted to the CCU are prone to serious risks due to rapid changes in their physical or mental conditions. In addition, the provision of care not tailored to the needs of the patients worsens their conditions (Yeh et al., 2010). One of the most important needs of such patients is to gain a proper and comprehensive attitude towards both the disease and the quality of care they receive (Pakdaman et al., 2019).

According to the Institute of Medicine, quality healthcare is defined as providing safe, timely, effective, efficient, fair and patient‐centred care (PCC; Institute of Medicine Committee on Quality of Health Care in America, 2001). The PCC is described as an innovative approach towards planning, provision and evaluation of healthcare and is based on mutually‐beneficial cooperation among patients, families and healthcare providers (Randall et al., 2020). Studies have reported that PCC is effective in achieving the triple aims of health system reforms designated by the Institute of Medicine (i.e., improving the patient's and family's experience of care, including quality and satisfaction, improving health outcomes and reducing the per capita costs of healthcare; Goldfarb et al., 2017; Kiwanuka et al., 2019; Kogan et al., 2016; Mitchell et al., 2016; Riley et al., 2014). Despite the growing recognition of the importance of PCC and its efficacy, studies have shown that patients suffer from a few important problems, such as the lack of access to important information, poor understanding of therapeutic options, receiving no explanations about the drugs and receiving no responsive and supportive services from their caregivers (Araki, 2019; Malhotra, 2015).

The presence of gaps between healthcare providers and patients has a negative effect on the acceptance of treatment, treatment costs and patients' outcomes (Malhotra, 2015). The results of studies in Iran also show that the achievement of patients' complete satisfaction with nursing care has not been possible. In addition, the results of the aforementioned studies demonstrate that the quality of nurses' work in the critical care unit was moderate because they used only 15%–20% of their potential efficiency at work (Esmaeili et al., 2014; Ghamarizare et al., 2008; Madani et al., 2010). The aforementioned investigations were quantitative studies that were conducted without considering barriers to PCC. Therefore, they cannot deeply explore the levels of experience, especially those of the involved individuals.

In addition, to increase the quality of care, accreditation has been considered a requirement by the Ministry of Health for the evaluation of hospitals in Iran since 2012. Accreditation is a systematic non‐organizational evaluation process performed to improve the quality and safety of care. By providing standards, this process leads hospitals to create a comprehensive, systematic management system and promote a safe, quality and patient‐centred culture (Ghane & Esmaeili, 2019; Rassouli et al., 2020). However, the results of some studies revealed that Iran's health system faces challenges in the accreditation process, including PCC (Asefzadeh & Navvabi, 2017; Ghane & Esmaeili, 2019; Rassouli et al., 2020). In the process of accreditation of hospitals, PCC does not have a clear, specified and integrated status. Despite the achievement of the first and second ranks in accreditation, the major problem of the health system is the issue of quality and patient‐centred services (Ghane & Esmaeili, 2019).

The implementation of PCC can vary depending on the patient population, healthcare providers, environment and professionals' and patients' understanding of the items comprising care (McCance et al., 2009; Moore et al., 2017). McCormack states that the context of the care environment has the greatest potential to limit or support PCC in practice (McCormack, 2004). One of the contexts in which caring and the nurse's role are likely to have changed and expanded is cardiac intensive care. Cardiac intensive care today offers a comprehensive management strategy for the treatment and evaluation of patients with a variety of heart diseases. Changes and developments in cardiac intensive care might also imply changes and development in nursing and consequently in caring (Andersson et al., 2015). It is noteworthy that patient‐centred critical care units have always attracted the attention of professional organizations for critical care nursing and medicine as suitable contexts for providing patient care (Kiwanuka et al., 2019). In such a model, the patients benefit from necessary training and support for decision‐making and contribution to self‐care (Hammerich et al., 2019).

A review of the literature indicates that the critical care context is more suitable for the provision of PCC, which might be due to the higher levels of care and observance of standards in critical care units (Esmaeili et al., 2014; Hobbs, 2009). However, the provision of care in such areas is not easy, and it is necessary to have healthcare providers highly qualified in providing quality care (Esmaeili et al., 2014). Andersson et al. (2015) described nurses' experiences of caring in the coronary care unit (CCU) as operating somewhere between the nurses caring values and the contextual conditions in which caring occurred. This issue challenges their ability to sustain caring in accordance with their values and patients' preferences (Andersson et al., 2015).

Despite great efforts to practice PCC, most healthcare systems are faced with challenges in the effective implementation of PCC (Santana et al., 2018). A literature review showed that a few studies have reported on barriers to PCC in the critical care unit (Downey et al., 2006; Esmaeili et al., 2014; Ganz & Yoffe, 2012; Moore et al., 2017; Riley et al., 2014; Turnbull et al., 2016; Van Mol et al., 2017). The barriers reported in the aforementioned studies included lack of understanding of PCC, daily rushed workloads, visiting policy, time constraints, competing priorities, difficult patients, healthcare providers' attitudes, interdisciplinary barriers (Downey et al., 2006; Esmaeili et al., 2014; Ganz & Yoffe, 2012; Moore et al., 2017; Riley et al., 2014; Turnbull et al., 2016; Van Mol et al., 2017). Most of the aforementioned studies have been performed in intensive care units. Therefore, considering that PCC is a multidimensional and context‐based concept (Esmaeili et al., 2017; Hudon et al., 2012), understanding the barriers to the implementation of PCC in the CCU can be helpful in the identification of strategies needed for its successful implementation and sustainability. The current study aimed to explore the barriers to the implementation of PCC in the CCU based on the experiences of patients, nurses, physicians and nursing managers.

## 2 METHODS

This qualitative study is part of larger research (a PhD thesis in nursing), entitled ‘Improving the Quality of Nursing Care in the CCU Based on the Concept of PCC: An Action Research Approach’. The current study presents the results of the exploratory phase that aimed to explore the barriers to the implementation of PCC in the CCU from Iranian patients', nurses', physicians' and nursing managers' perspectives. Descriptive qualitative studies allow for the description or exploration of various aspects of phenomena, problems or issues. Descriptive qualitative studies can be used to answer questions about clinical workers' or health policymakers' concerns, experiences, knowledge, attitudes, perceptions, views and feelings and the barriers and facilitators to a given particular phenomenon (Sandelowski, 2000).

### 2.1 Setting and participants

The setting for the present study was a CCU of a teaching hospital affiliated with Abadan University of Medical Sciences, Khuzestan, Iran. The nurse‐to‐patient ratio was 1:3 in the morning shift and 1:4.5 in the evening and night shifts.

For the enrichment of the data, different participants (i.e., patients with maximum diversity in terms of age, gender and educational, cultural, economic and social levels and nurses, physicians and nursing managers in terms of work experience) were selected based on purposeful sampling method. In this method, the researcher was looking for individuals with rich experiences about the phenomenon and the ability and desire to express them. In purposeful sampling, the goal for qualitative researchers is to provide a detailed or in‐depth description of the culture or phenomenon. The reason for choosing this sampling method was that it ensures obtaining rich data in qualitative studies (Palinkas et al., 2015; Speziale et al., 2011).

The inclusion criteria set for the patients were hospitalization at the CCU at least for 3 days, awareness and willingness to participate in the study and appropriate physical conditions to answer the questions and share their experiences with the researcher. The inclusion criteria defined for the healthcare providers were at least 2 years of clinical experience, facilitation of understanding of local contextual factors affecting PCC and willingness to share their experiences with the participants under the study. For sampling, the researcher in different shifts referred to the CCU of a teaching hospital and started to interview a critical care nurse and then continued the interviews with other key informants in this field. A total of 29 participants, including 10 CCU nurses, one assistant nurse, two cardiologists, nine nursing managers and seven CCU hospitalized patients, participated in this study. Sampling continued up to reaching data saturation. Therefore, sampling stopped when the data obtained in interviews were duplicated, and the research question was answered (Speziale et al., 2011).

### 2.2 Data collection

Data collection was carried out from April 2019–August 2020 through individual, face‐to‐face, in‐depth and semi‐structured interviews. Each interview lasted for 40–70 min on average. All interviews were conducted by the first researcher in a quiet environment in the unit. Initially, an interview guide (Table 1) was developed based on the research question and the existing literature. The guide included open‐ended questions about the barriers to the implementation of PCC in the CCU. The content validity of the guide was assessed by a CCU nurse with 10 years of professional experience and two faculty members of a critical care department who were experienced in both critical care and qualitative studies. Then, the first author conducted two pilot interviews using the guide. The pilot interviews were evaluated by the second and third authors and confirmed the guide. The interviews began with a general, open‐ended question (the interview guide). Then, based on the participants' answers and according to the purpose of the study, the interviews took a more structured form using clear questions, and the challenges of PCC in the CCU were addressed. The participants were asked to cite concrete examples. Additionally, exploratory questions, such as ‘What do you mean?’, ‘Please explain more about this?’ or ‘My impression of what you are saying…, did I get it right?’, were used to give depth to the interview. The analysis of the data from each interview was a guide for the next interview. The interviews were recorded. Data saturation was achieved when no new codes or findings were obtained.

**TABLE 1:** Sample questions to describe challenges in implementation of patient‐centred care in cardiac care unit

Participants	Questions
Nurses and physicians	What kind of care services do you provide for patients with cardiovascular disease?
	What care services provide satisfaction for these patients?
	In your experience, what are the most important barriers to the implementation of PCC in the CCU?
Patients	Describe your experiences with how the work was performed and how the care you received was in this unit?
	What were the services that did not meet your expectations, and what were your expectations that were not met? Please explain.
Mangers	What are your criteria for controlling, monitoring and evaluating the quality of nursing care in the CCU?
	Based on your experience, what are the challenges to the implementation of PCC in the CCU?

Abbreviations: CCU, cardiac care unit; PCC, patient‐centred care.

### 2.3 Analysis

In this study, the conventional content analysis method was used to analyse the data. The analysis of qualitative data was performed based on the proposed steps by Lundman and Graneheim (Graneheim & Lundman, 2004). In the beginning, the first and second authors frequently listened to the interviews and read the interview transcripts to immerse in the data and gain a sufficient understanding of them. Then, the text of the interview was written and read word for word. Afterwardsfv, each transcript was independently analysed. The units of meaning and the units of condensed meaning were extracted from the first and second interviews. The extracted units of meaning were then discussed to reach a general consensus. In the next step, the first author analysed the remaining 27 interviews. At the beginning of each interview, the text was read several times to gain a full understanding of it. The text was then divided into semantic units. Each semantic unit was converted to a compact semantic unit and labelled with an appropriate code. The initial codes were then categorized based on their similarities and differences and placed in subcategories. Finally, the subcategories were combined to form the main classes. Coding and classification were supervised by the second and third authors.

### 2.4 Trustworthiness

The four criteria proposed by Lincoln and Guba were considered to achieve the accuracy and stability of the data (Polit & Beck, 2017). For the increase in the validity, reliability and dependability of the data, the issues, such as allocating sufficient time to collect data, tracking back and forth frequently between the data and analysis and maintaining long‐term involvement, were observed, which increased the breadth and depth of the data. The results of the analysis were provided to three faculty members to assure the credibility, dependability and confirmability of the findings. Purposive sampling with maximum variability was used, which contributed to the generalizability of the findings. In addition, the researcher tried to increase the credibility of the research by sufficient involvement and interaction with the participants and by the collection of valid information.

### 2.5 Ethics

The present study was approved by the Ethics Committee of Ahvaz Jundishapur University of Medical Sciences, Khuzestan, Iran (ref no. IR.AJUMS.REC.1397.813). Permission to carry out the study was received from the Vice‐Chancellor for Research, Abadan University of Medical Sciences. The objectives and the method of the study were presented to the participants. Moreover, informed consent was obtained from the patients. The participants were assured of the confidentiality of their information and gave permission to tape‐record the interview.

## 3 RESULTS

The participants included seven patients with cardiovascular disease and 22 healthcare providers (Table 2). In this study, three main categories and eight subcategories were extracted as challenges in the implementation of PCC in the CCU, which are briefly presented in Table 3.

**TABLE 2:** (a) Demographic characteristics of patients. (b) Demographic characteristics of care providers

(a)						
Participant	Age (year)	Gender	Literacy level	Marital status	Occupation	History of affliction (year)
Patient 1	71	Female	Primary school	Married	Retired	10
Patient 2	46	Male	High school	Married	Laborer	4
Patient 3	42	Male	BSc	Married	Employee	2
Patient 4	64	Female	Primary school	Married	Housekeeper	8
Patient 5	45	Male	High school	Married	Free job	3
Patient 6	57	Male	High school diploma	Married	Retired	5
Patient 7	55	Female	High school diploma	Married	Housekeeper	4

(b)				
Work experience	Literacy level	Gender	Age (year)	Participant
15	BSc	Female	42	Nurse 1
9	BSc	Female	36	Nurse 2
28	BSc	Female	52	Nurse 3
20	BSc	Female	44	Nurse 4
20	BSc	Female	46	Nurse 5
14	MSc	Female	37	Nurse 6
15	BSc	Female	38	Nurse 7
13	BSc	Female	39	Nurse 8
14	BSc	Female	36	Nurse 9
22	BSc	Female	46	Nurse 10
10	High school diploma	Male	40	Nurse assistant
23	BSc	Female	54	Head nurse
25	BSc	Female	53	Matron
21	MSs	Female	45	Educational supervisor
18	BSc	Female	41	Clinical supervisor 1
12	BSc	Female	37	Clinical supervisor 2
20	BSc	Female	45	Clinical supervisor 3
18	MSc	Female	42	Health promotion supervisor
3	Cardiologist	Male	35	Physician 1
10	Cardiologist	Male	45	Physician 2
5	BSc	Female	30	Accreditation manager
12	BSc	Female	37	Deputy matron

**TABLE 3:** Challenges in implementation of patient‐centred care in cardiac care unit: categories, subcategories and codes

Categories	Subcategories	Codes
Challenges related to the organization	Lack of a suitable context to provide PCC	Shortage of manpower Insufficient and inadequate equipment Inefficient organization of nursing care Lack of guidelines and practical models Limited learning resources and lack of a role model Weakness in staff training
	Task‐oriented healthcare system	Implementation of imperative care due to fear of the law Provision of task‐based care Existence of imposed activities
Challenges related to healthcare providers	Lack of a holistic view toward care	Dominance of medical vision in the health system traditional approach toward clinical practice Poor care performance
	Patient‐centeredness as the missing link in care	Weakness in the continuity of care Inappropriate attitude of staff toward PCC Weakness in patient recognition and evaluation
	Ineffective professional interactions	Undesirable professional relationships
	Low job motivation	Feeling of worthlessness Low salaries and benefits Insufficient support from managers Lack of professional independence
Challenges related to patients	Lack of patient's health literacy	Lack of knowledge about self‐care activities Patient's inappropriate attitude toward chronic disease Lack of understanding of the importance of adherence to treatment
	Exposure to and acceptance of paternalism	Adherence to decisions made due to lack of knowledge and self‐confidence Paternalism relationship

Abbreviation: PCC, patient‐centred care.

### 3.1 Challenges related to the organization

Organization means the context in which services are provided and is a prerequisite for facilitating processes and influencing outcomes to achieve PCC (Charosaei et al., 2021). This category included a lack of a suitable context to provide PCC and the task‐oriented healthcare system.

#### 3.1.1 Lack of a suitable context to provide PCC

Providing nursing care in the CCU requires the provision of special conditions. The participants believed that the lack of a proper context was one of the challenges in the implementation of PCC. The factors, such as the shortage of manpower, insufficient and inadequate equipment, inefficient organization of nursing care, lack of guidelines and practical models, limited learning resources, lack of a role model and weakness in staff training, were some examples of this subcategory. The participants considered the weakness in staff training and holding specialized intensive care courses as one of the prominent barriers to the provision of appropriate conditions. The participants stated that specialized training in cardiac care was not enough, and in‐service training was not based on staff needs assessments. Nevertheless, every nurse needs continuous specialized training for professional and scientific growth. In this regard, one of the nurses with 15 years of working experience said:

As a critical care nurse, I should not work on the basis of practical and experimental knowledge. I should receive ongoing training in cardiovascular disease cases, arrhythmias and new evidence‐based information. However, the authorities have not held any classes for almost more than a year. (Nurse no. 7)

The participants also stated that although the concept of PCC had been considered in the hospital's missions and values and in the accreditation process, there were no clear guidelines for applying this concept in practice. The participants believed that the role models were useful for the formal and informal education of nurses in clinical settings and put nurses in a position where they can share their knowledge of care patterns with peers. A nurse with 22 years of working experience said:

We need role models that inspire us, and it should be a model that we can apply to our day‐to‐day care based on our patient's condition. (Nurse no. 10)

The ineffective organization of nursing care due to the inefficient supervision and poor management of nursing care were other factors negatively affecting the provision of an appropriate context. One of the nurses with 14 years of working experience stated:

The system does not care whether you pay attention to the patient's mental needs or not. The goal is just to do the tasks. Now, when the supervisor comes to the ward, s/he asks a series of questions. For example, s/he asks what the diagnosis of the patient is. The emphasis is more on the documents than on the patient. (Nurse no. 9)

The shortage of manpower, facilities and equipment was another problem experienced by most healthcare providers. Concerning the shortage of manpower, one of the nurses with 9 years of working experience said:

When the patient‐to‐nurse ratio is high, the nurse's focus on care is decreased, and many of the nursing practices may be missed. (Nurse no. 2)

#### 3.1.2 Task‐oriented healthcare system

The participants believed that the culture of hospitals was highly physician‐centred and based on routine and division of tasks. Nurses are directly and indirectly limited by the effects of this culture. Based on the participants' experiences, the patients' unstable conditions, the system's emphasis on task‐based care and the fear of being reprimanded were the factors that limited the participants to provide PCC. A nurse with 22 years of working experience said:

This is a critical care unit. Suddenly, a patient's condition may worsen, and you have to go to his/her bed quickly …, so what about other patients? I just think that I should finish my work quickly so that I will not be reprimanded tomorrow. I did my best not to miss any orders. (Nurse no. 10)

In addition, limited time and lack of knowledge about the professional role caused nurses to focus on task‐centred care instead of PCC and tend to follow the physician's orders. On the other hand, the focus of the authorities on documentation had made physical care a priority.

Nurses don't talk to us very much; they only talk when they come to give our medicine or change our intravenous tubes. For example, three nurses come, look, write down the files, and leave the room. They do not care at all that are you ok or not? Nurses do not have time to answer our questions at all. (Patient no. 7)

The imposed activities, ranging from care to non‐care ones, were almost an issue that nurses repeatedly addressed as one of the barriers. When the nurse is doing or pursuing activities other than his/her tasks, there would be no time to interact with the patients.

### 3.2 Challenges related to healthcare providers

This category included a lack of holistic view towards care, patient‐centeredness as the missing link in care, ineffective professional interactions and low job motivation.

#### 3.2.1 Lack of a holistic view towards care

A holistic view is one of the most important aspects of nursing care, and the patient cannot be considered without paying attention to his/her totality. This subcategory features the dominance of medical vision in the health system, the traditional approach towards clinical practice, and poor care performance.

The participants believed that the shortage of manpower, managers' lack of time and interest in physical care and emphasis on the provision of holistic care led to poor care performance. In such situations, care is more focused on satisfying the patient's physical aspects; as a result, the patient's psychological, spiritual, emotional and social aspects are ignored by the nurses.

Support from the managers is very important. For example, they ask us to control the patient's vital signs, to chart, but not to communicate with, or educate, patients… (Nurse no. 9)

Additionally, the traditional approach to clinical practice has made the physician's orders, nurse's diagnoses and routine tasks the basis of the care. The participants also stated that the factors, such as a technology‐based perspective, performing repetitive tasks without considering the patients' real needs and the absence of specific nursing models, which all result from biomedical dominance in the health system, have led the healthcare providers to see the patient as a disease rather than a unique individual on his/her own.

Unfortunately, most of our criteria for evaluating a patient are objective data and the monitoring screen, and other issues, such as the likely problems of the patient, are not attended. (Nurse no. 3)

#### 3.2.2 Ineffective professional interactions

Deficiencies in the relationship between the nurse and other members of the treatment team will create an unhealthy work environment and lead to negative consequences for patients, which also include undesirable professional relationships. The participants attributed the inability to establish an effective two‐way relationship to the lack of training in communicative skills, low self‐esteem and nurses' non‐acceptance of the role of the counsellor and coordinator in the treatment team.

We have little courage to comment on communication because we have not been trained. We must communicate with awareness and power. Others need to know that I can provide treatment consultations. In the treatment team, coordination is the responsibility of the nurse. (Nurse no. 5)

One participant with 28 years of working experience described the unfavourable professional relationship as follows:

Because we have not learned to work as a team, each team does its own duty. Physicians, regardless of the organization, issue their own orders according to the patient's physical problems and nurses only try to perform these orders, which makes many processes inefficient. However, in general, we should see ourselves as a team, and not as individuals separate from the team. (Nurse no. 3)

#### 3.2.3 Low job motivation

While motivation is an important factor for continuing one's efforts, losing motivation will prevent the emergence of individuals' capabilities and reduce the quality of services they provide. Feeling of worthlessness, low salaries and benefits, insufficient support from managers and lack of professional independence were the characteristics of this subcategory. The participants stated that not being seen by superiors, lack of support from managers and failure in their efforts led to their frustration and annoyance.

You see that the patient has a cardiac arrest, and you make every effort, but the patient does not return. The supervisor comes and says your team worked poorly. Then, you feel exhausted. They see nurses accountable for everything. You feel that your efforts are useless. (Nurse no 9)

Regarding the low salaries and benefits, one of the participants with 5 years of working experience said:

The payment is not good, and fee‐for‐service is not paid regularly, so our motivation is reduced. (Head of the Accreditation Committee)

The participants also attributed the decline in motivation to a lack of freedom of action and deprivation of the right to make decisions.

#### 3.2.4 Patient‐centeredness as the missing link in care

The dominance of traditional methods and adherence to routine is the reference for the patient's needs; therefore, less attention is paid to the patient's values, preferences and concerns. The weakness in care continuity, the inappropriate attitude of staff towards PCC and the weakness in the careful examination and recognition of the patient were the characteristics of this subcategory. Based on the participants' experiences, the factors, such as the indifference of the treatment team to rehospitalization, lack of a pursuing healthcare system and lack of discharge planning in the hospital, lead to weakness in the continuity of care.

Of course, some heart failure patients with low ejection fraction come back, no matter what you do, because their disease has progressed. However, follow‐up with these patients is important because they are at different stages and need self‐care. Many problems may occur to them at home. Therefore, both the patient and their families need counselling and education. For example, what should we do if s/he has shortness of breath…? The nurse can prevent the complications of repeated hospitalization with a guide and telephone call. (Nurse no. 5)

The inappropriate attitude of the staff towards PCC was also mentioned as one of the barriers. For example, case method nursing meant non‐cooperation and inattention to the patient, distance from other patients and interference with nursing work to nurses. The participants also stated that accurate patient evaluation had an important role in the implementation of PCC and could lead to the individualization of care based on the unique characteristics of each patient.

### 3.3 Challenges related to patients

The participants attributed patients' inappropriate attitudes towards chronic diseases and lack of understanding of the importance of adherence to treatment to patients' low levels of health literacy, making patient education difficult for the staff. Moreover, lack of sufficient knowledge about their condition and feeling unable to adapt to problems due to the disease led to the passivity of the patient in care. This category included two subcategories the lack of patient's health literacy and the exposure to and acceptance of paternalism.

#### 3.3.1 Lack of patient's health literacy

In this study, the participants believed that inadequate health literacy was a serious barrier to the patient's involvement in care, negatively affecting disease management and patient involvement in self‐care activities.

Some patients' problems are due to lack of adherence to treatment and diet because they do not know what to do due to lack of knowledge. Some of them do not know that their disease is chronic, and they should always be monitored. They think they will get better after a while and do not accept permanent changes in their lifestyle. (Nurse no. 8)

#### 3.3.2 Exposure to and acceptance of paternalism

The participants' experiences showed that when a patient entered the hospital, s/he encountered a paternalistic relationship and acted according to what others had said. Following such decisions, which result from the lack of knowledge, the lack of self‐confidence and the presence of paternalism, is the characteristic of this subcategory.

Because I have no information about my disease and my conditions and I do not know anything about my treatment, I have left everything to the physicians and nurses. Some physicians and nurses do not answer clearly when I ask them something. They do not have enough time to explain to each patient individually. So, I should accept what they say because I have no information, but they have a lot of information, and they are specialists. (Patient no. 7)

## 4 DISCUSSION

This study was performed on the barriers to the implementation of PCC in the CCU based on the experiences of patients and healthcare providers. These barriers fall into three main categories, including challenges related to patients, challenges related to healthcare providers and challenges related to the organization. The lack of a suitable context to provide PCC was one of the challenges related to the organization. The achievement of PCC and increase in patient satisfaction require the availability and optimization of its due context (Esmaeili et al., 2017).

In the present study, the participants addressed the shortage of manpower as one of the reasons for the lack of a suitable context in which PCC should be provided. They stated that due to the high workload and unpredictability of the patients' conditions, they were only able to perform routine care. Similarly, Nobahar revealed that the right number of nurses was an important factor in the achievement of better outcomes for patients and nurses. When the number of nurses was low, they provided only the necessary care (Nobahar, 2014; Schubert et al., 2013). In a qualitative study, Torabizadeh et al. (2013) also pointed to the violation of the patient's dignity due to nursing shortage. They stated that the nursing shortage and the increase in nurses' workload were the most important challenges in the healthcare system, reducing the quality of services, preventing the on‐time conduction of many care practices and consequently violating the patient's dignity (Torabizadeh et al., 2013).

Inadequate and inappropriate equipment was another factor affecting the care context. In this study, several factors, such as the lack of pharmaceutical stock, failure of infusion pumps and lack of equipment and facilities were mentioned. The aforementioned findings are in line with the results reported by Esmaeili et al. (2014) that mentioned lack of care equipment and financial resources as one of the barriers to PCC achievement. Pretorius and Klopper also reported that appropriate equipment and technology were the key components of a healthy critical care unit environment. They demonstrated that the presence of appropriate equipment reduced nurses' work problems and improved the quality of nursing care (Pretorius & Klopper, 2012).

Lack of guidelines and practical models and weakness in staff training in specialized cardiac care were addressed as other barriers associated with the non‐provision of appropriate care context. The participants stated that specialized training for cardiac care was not sufficient, and the training provided for nurses working in the CCU was not based on the assessment of their needs. Ahmadi et al. (2011) showed that proper in‐service training had a valuable role in the education of professional nurses and could play an important role in improving the quality of nursing care. Esmaeili et al. (2014) also reported that the mere introduction of a series of concepts without addressing their nuances would prevent a comprehensive and complete achievement of the defined goal. The development of specific instructions and guidelines appropriate to the conditions, facilities and human resources in the ward is one of the duties of organizational managers to clarify the path to PCC and achieve patient satisfaction. Becoming familiar with the instructions, concepts and nursing job descriptions is necessary for achieving this objective (Esmaeili et al., 2014).

The participants stated that paying attention to role models was very important in the formation of PCC. More importantly, they believed that recognizing and highlighting such models played a prominent role in the success of other individuals' role models and their performance. Esmaeili et al. (2014) also showed that the existence of effective and efficient models played an important role in guiding performance and providing nursing care. The presence of successful and exemplary colleagues in the workplace environment and using their experience and knowledge in guiding nurses towards PCC were beneficial (Esmaeili et al., 2014).

The ineffective organization of nursing care was another barrier related to the lack of care context, which included the phenomena, such as weakness in the management of nursing care and inefficient supervision. Rafiee et al. (2005) showed that employees were strongly influenced by the organization, and the employees found managers incapable of performing their duties. They reported that there was not enough supervision over care, which turned into a problematic issue in the evening and night shifts. The head nurses believed that multiple duties did not allow them to supervise properly, and both groups were under pressure in this regard. The authors stated that lack of adequate supervision reduced the opportunity for proper evaluation; as a result, the true value of the staff's activities and capabilities was not recognized. Accordingly, the emotional stress and lack of motivation were experienced by the employees, reducing the quality of care (Rafiee et al., 2005).

The task‐oriented healthcare system was another barrier related to the organization. The participants attributed the task‐centred healthcare system to several factors, such as the implementation of imperative care due to fear of the law, the provision of task‐based care and the compulsion to perform imposed activities. Deploying a task‐based healthcare system leads to skill depletion, reduced communication with the patient and ignorance of the patient's totality. In this system, in most cases, each employee performed his/her duties, and some care was missed. In these conditions, the goal was to do the job regardless of the actual care needs, quality and outcomes. In this regard, Strandås et al. (2019) demonstrated that the provision of formal and task‐centred services resulted in the ignorance of the professional judgement of nurses in healthcare organizations; consequently, nurse–patient interaction, unique personal care and care could be impaired.

The second main category of challenges was related to healthcare providers. This category included a lack of a holistic view towards care, patient‐centeredness as the missing link in care, ineffective professional interactions and low job motivation. According to the participants, the lack of a holistic view towards care in the CCU was due to the priority which is given to medical problems and technology used to save patients' lives by healthcare providers. The participants argued that the dominance of medical vision in the health system, the traditional approach to clinical practice and the poor care performance challenged the maintenance of holistic nursing care. In this regard, Cheraghi et al. (2017) showed that providing care based on the biomedical model was in conflict with PCC. Cheraghi et al. (2017) also concluded that the integration of biomedical models with the humanistic approach in the caregiver and the emphasis on the psychosocial and spiritual aspects of care were the prerequisites of PCC. In Moore et al.'s study, the traditional structure and activities were identified as one of the barriers to PCC. The participants of the aforementioned study reported that working based on the traditional care system restricted their freedom in performing activities different from routine care (Moore et al., 2017).

The results of this study demonstrated that a feeling of worthlessness, low salaries and benefits, insufficient support from managers and lack of professional independence were the factors that reduced the participants' job motivation. In another study, Jafaraghaee et al. (2015) showed that the lack of attention of authorities to the suggestions and supervision of nurses and the lack of support from managers and head nurses in emergencies led to dissatisfaction, low productivity and poor consequences in the care provided for patients. Similarly, Nasrabadi pointed out the lack of motivation and mere obedience of nurses to the physician's orders. The current role of these nurses as doers of routine and sometimes extra‐professional tasks is in conflict with their professional role as caregivers who should recognize and prioritize the problems, plan, intervene and evaluate on the basis of their knowledge (Nasrabadi et al., 2004).

Lack of coordination between members of the care team, lack of teamwork and poor intra‐ and interprofessional communication were among the issues mentioned by the participants as the factors negatively affecting professional interactions. The results of various studies indicated that team inconsistencies led to serious concerns in achieving quality in caregivers (Esmaeili et al., 2014; Randall et al., 2020). Similarly, the results of this study showed that team inconsistencies led to numerous problems for patients, including late discharge or difficulty in receiving timely and required information. Coordination in care settings occurs at different levels, namely among healthcare workers, patients and their families. Meanwhile, coordination among health workers plays an important role in achieving PCC and increasing patient satisfaction (Bodenheimer, 2008). In their study, Esmaeili et al. (2014) mentioned that disagreement between health workers, especially physicians, and team inconsistencies were barriers to achieving PCC.

The findings of the present study indicated a shortage in the implementation of PCC due to the weakness in the continuity of care, the inappropriate attitude of staff towards PCC and the weakness in patient recognition and evaluation. In this regard, Joolaee et al. (2008) showed that this was the case in Iran due to the disease‐centred health system. In other words, lack of knowledge about patient's rights, lack of experienced and skilled personnel, inappropriate management policies, lack of monitoring systems, lack of accountability, lack of patient‐centred services, lack of written job descriptions and lack of work conscience were among the important barriers to respecting the patient's rights and considering the patient himself/herself rather than his/her disease (Joolaee et al., 2008). Moore et al. (2017) stated that in systems with poor continuity of care, healthcare providers had a limited and task‐based view towards the individual's disease and ignored the patient's totality, leading to role divisions and care fragmentations. Role divisions and care fragmentation limit the opportunities for health professionals to see a patient's progress and give them only limited exposure to the complete course of a patient's illness (Moore et al., 2017).

Another barrier to the implementation of PCC was patient‐related challenges. Patients accepted paternalistic communication and lack of involvement in care due to being overshadowed by the disease, lack of sufficient knowledge about their condition and feeling unable to cope with problems. In this regard, Larson believes that facing self‐disability is an important aspect of how individuals respond as patients in a hospital setting. Patients have to deal with the disease and cope with the role of an individual in the unknown environment of the hospital. Most patients feel vulnerable and out of control. In other words, patients might become dependent and passive (Larsson et al., 2011).

According to the participants, lack of health literacy on the part of patients was also one of the challenges related to patients. Lack of knowledge about self‐care activities, patients' inappropriate attitude towards chronic disease and lack of understanding of the importance of adherence to treatment were some of the issues reported in this study. In their study, Williams et al. (2002) also stated that inadequate health literacy was a serious barrier to performing self‐care behaviours. Similarly, Morrow et al. (2006) revealed that low health literacy was associated with poor health status, less self‐care knowledge, less adherence to treatment, higher rates of rehospitalization and increased care costs.

### 4.1 Limitations

The selection of participants from one CCU of a teaching hospital could limit the generalizability of the obtained findings. Efforts were made to recruit participants with maximum variations in terms of age, gender and educational, cultural, social and economic levels in patients and professional experience in nurses, physicians and nursing managers to overcome the aforementioned limitation. Therefore, it is suggested to perform further studies in the CCU of different private and teaching hospitals. In this study, only in‐depth interviews were used to collect the data. Therefore, it is suggested to use a focus group interview consisting of a sample representative to obtain more depth of information.

## 5 CONCLUSION

This study examined the barriers to the implementation of PCC in the CCU from the perspective of patients and healthcare providers. The findings suggest that the current care plan needs to be reviewed and enhanced. It was also shown that these barriers were very diverse, complex and interrelated and included a wide range of challenges related to the organization, healthcare providers and patients. Therefore, the provision of a suitable context, interprofessional education, modification of clinical guidelines to empower care providers to involve patients in the care process, personalization of care, promotion of interprofessional cooperation and reduction of occupational problems were some of the issues that should be considered while planning for and implementing PCC.

## CONFLICT OF INTEREST

There are no conflicts of interest.
